# The Use of Chemical-Chemical Interaction and Chemical Structure to Identify New Candidate Chemicals Related to Lung Cancer

**DOI:** 10.1371/journal.pone.0128696

**Published:** 2015-06-05

**Authors:** Lei Chen, Jing Yang, Mingyue Zheng, Xiangyin Kong, Tao Huang, Yu-Dong Cai

**Affiliations:** 1 College of Life Science, Shanghai University, Shanghai, 200444, People’s Republic of China; 2 College of Information Engineering, Shanghai Maritime University, Shanghai, 201306, People’s Republic of China; 3 Institute of Health Sciences, Shanghai Institutes for Biological Sciences, Chinese Academy of Sciences, Shanghai, 200031, People’s Republic of China; 4 Drug Discovery and Design Center, Shanghai Institute of Materia Medica, Shanghai, 201203, People’s Republic of China; Seoul National University, REPUBLIC OF KOREA

## Abstract

Lung cancer causes over one million deaths every year worldwide. However, prevention and treatment methods for this serious disease are limited. The identification of new chemicals related to lung cancer may aid in disease prevention and the design of more effective treatments. This study employed a weighted network, constructed using chemical-chemical interaction information, to identify new chemicals related to two types of lung cancer: non-small lung cancer and small-cell lung cancer. Then, a randomization test as well as chemical-chemical interaction and chemical structure information were utilized to make further selections. A final analysis of these new chemicals in the context of the current literature indicates that several chemicals are strongly linked to lung cancer.

## Introduction

With more than one million cases per year worldwide, lung cancer causes significantly more mortalities than do other cancers [1]. Furthermore, due to delayed diagnosis, the overall 5-year survival rate remains at only 15% [2]. Based primarily on histological considerations, lung cancer can be categorized as either non-small lung cancer (NSCLC) or small-cell lung cancer (SCLC), with the former accounting for approximately 85% of cases. The NSCLCs are divided into three subtypes: adenocarcinoma, squamous-cell carcinoma and large-cell carcinoma. The first two subtypes comprise 90% of NSCLC cases [3,4].

There are various molecules that participate in tumorigenesis and treatment, most of which function by affecting the driver mutation genes. Additionally, some exotic or synthetic molecules have been used as effective drugs in chemotherapy. The standard platinum doublet chemotherapeutic has been used to effectively treat NSCLC [2]. It has been observed that epidermal growth factor receptor (EGFR) mutations are associated with approximately 15% of NSCLC patients, and administration of gefitinib, a selective chemotherapeutic agent targeted at EGFR, led to longer patient survival [5,6,7,8]. In the second-line treatment for SCLC, administration of topotecan, a camptothecin-based drug, improved the survival of patients from 14 to 26 weeks [9,10]. HER2, also known as ERBB2 (erb-b2 receptor tyrosine kinase 2), is a receptor tyrosine kinase, which is overexpressed in more than 20% of NSCLCs and mutated in approximately 2% of NSCLCs [11,12,13,14]. In clinical trials, BIBW 2992 was assessed for NSCLC treatment and was shown to be effective for patients with lung adenocarcinoma [15]. PIK3CA, a member of the phosphatidylinositol 3-kinases, is a key mediator between growth factor receptors and the downstream signaling network [16]. Mutations in PIK3CA have been identified in approximately 2% of NSCLC cases, with particular enrichment at exon 9 [17,18,19]. In mice, BEZ235, a small molecule inhibitor, inhibited the growth of tumor cells by targeting PI3K and the mTOR protein and is being used in early clinical development [20].

In addition to the molecules involved in chemotherapy, many substances contribute to the complex process of carcinogenesis. At various stages, ion channels play key roles in tumorigenesis and lung cancer pathology. Ca^2+^ channels are associated with the pro-proliferative action of mitogen in lung cancer cell lines [21]. Increased expression has also been observed in Na^+^ and K^+^ channels in lung tumors [22,23]. However, the detailed mechanism is still unclear. Mg^2+^ is an important part of many essential enzymes involved in lung carcinogenesis such as TSLL2, which participates in cell adhesion [24]. Oxygen and oxidative stress function as messengers and regulators of cell proliferation, apoptosis and survival. DNA damage, including single/double-stranded DNA breaks and purine/pyrimidine modifications, are induced by reactive oxygen species (ROS). The lung is the major organ affected by environmental pollutants and endogenous ROSs. Chronic inflammation and activation of leucocytes, which generate high-dosage ROS and affect normal cell density, are induced by cigarette exposure [25,26]. Additionally, many other hazardous materials, including asbestos, arsenic and polycyclic aromatic carbohydrates, were identified as potential pathogenic factors in lung cancer [27].

Although some chemicals have a proven association with lung cancer, this knowledge is still limited compared with the quantity of newly discovered chemicals. Discovery of new chemicals that may influence the function of lung cancer is helpful to decrease the incidence of this disease and to design effective treatments. However, it is time-consuming and expensive to use traditional methods to detect new chemicals related to lung cancer because there are too many candidate chemicals to allow for a detailed analysis. Fortunately, the development of computer science provided an alternative screening method by introducing effective computational methods. Given the successful application of computer science to tackle various biological problems in many previous studies [28,29,30,31,32,33,34,35,36,37,38], we anticipate effective computational methods for the discovery of new candidate chemicals related to lung cancer.

Recently, Li *et al*. [39] proposed a computational method to identify new candidate genes in a protein-protein interaction network. This method can be easily generalized to identify candidate chemicals. In this study, the generalized method was applied to study two types of lung cancer: NSCLC and SCLC. We constructed a weighted network according to chemical-chemical interaction information retrieved from STITCH (Search Tool for Interactions of Chemicals) (latest version 4.0) [40,41]. To detect new chemicals related to lung cancer, we employed the known lung cancer-related chemicals retrieved from the CTD (Comparative Toxicogenomics Database) [42]. By applying a shortest path algorithm in the constructed network, we searched all shortest paths connecting any two known chemicals related to lung cancer. Chemicals occurring in any path were deemed candidate chemicals. Furthermore, a randomization test was executed to control false discoveries, and the interaction score provided in STITCH and compound similarity scores were employed to further screen chemicals that have strong links to lung cancer. Finally, we analyzed the relationship between the candidate chemicals and the two types of lung cancer. Interestingly, most of the candidate chemicals are potential chemotherapy drugs. The identification of multiple relevant molecules will improve the understanding and treatment of lung cancer.

## Materials and Methods

### 2.1 Chemicals related to lung cancer

The NSCLC and SCLC-related chemicals were downloaded from the CTD (accessed on June 19, 2014) [42] at the web sites http://ctdbase.org/detail.go?type=disease&acc=MESH:D055752&view=chem and http://ctdbase.org/detail.go?type=disease&acc=MESH:D002289&view=chem, respectively. In the CTD, the disease and chemical relationships were manually extracted from the literature. We only used chemicals with direct evidence of an association with NSCLC or SCLC, such as a marker, mechanism or therapeutic. After excluding chemicals without a record in the constructed network (see Section 2.2), 16 NSCLC-related chemicals and 13 SCLC-related chemicals were obtained (listed in **Table 1**). For convenience, let S_NSCLC_ and S_SCLC_ be sets consisting of 16 NSCLC-related chemicals and 13 SCLC-related chemicals, respectively.

**Table 1:** Chemicals related to two types of lung cancer.

NSCLC		SCLC	
PubChem ID	Name	PubChem ID	Name
CID2141	Amifostine	CID2907	Cyclophosphamide
CID2244	Aspirin	CID3690	Ifosfamide
CID3117	Disulfiram	CID3950	Lomustine
CID3121	Valproate	CID4168	Metoclopramide
CID3385	Fluorouracil	CID5426	Thalidomide
CID3690	Ifosfamide	CID5978	Indole Alkaloid
CID5426	Thalidomide	CID31703	Doxorubicin
CID5746	Mitomycin C	CID36462	Etoposide
CID36462	Etoposide	CID41867	Epirubicin
CID41867	Epirubicin	CID89594	Nicotine
CID72120	Nedaplatin	CID126941	Methotrexate
CID89594	Nicotine	CID5351344	Combretastatin A-4
CID91466	Matrine	CID5359596	Arsenic
CID126941	Methotrexate		
CID441207	Digitoxin		
CID5282379	Isotretinoin		

### 2.2 Construction of the weighted network

Some studies have shown that interactive chemicals (*i*.*e*., chemicals that can interact with each other) always share similar functions [29,31,43]. It is tempting to infer that known chemicals related to lung cancer have some common lung cancer-related functions. Thus, the interactive chemical of these chemicals also likely shares these functions. To investigate this possibility, we constructed a weighted network from chemical-chemical interactions data. These data were downloaded from STITCH (version 4.0, http://stitch.embl.de/) [40,41], a large scale database consisting of known and predicted interactions of chemicals and proteins, which are derived from experiments, databases and the literature. In the obtained file (chemical_chemical.links.v4.0.tsv.gz), each interaction contains two chemicals and one score that were obtained by integrating various information, including structures, activities, reactions, *etc*., thereby widely noting the associations between chemicals. In the calculations, the score of the interaction between the chemicals *c*_1_ and *c*_2_ is noted as *S*_*i*_(*c*_1_, *c*_2_). In particular, if the chemicals *c*_1_ and *c*_2_ do not occur as an interaction in the obtained file (chemical_chemical.links.v4.0.tsv.gz), *S*_*i*_(*c*_1_, *c*_2_) is set as zero. Additionally, to reduce the search space, we only considered the interactions between chemicals that have records in KEGG [44].

The constructed network interpreted chemicals as nodes. Two nodes were connected by an edge if and only if the corresponding chemicals interacted. Additionally, to utilize the fact mentioned in the above paragraph and using the shortest path algorithm to identify new candidate chemicals, each edge was assigned a weight defined by 1000- *S*_*i*_(*c*_1_, *c*_2_), where *c*_1_ and *c*_2_ were two corresponding chemicals of the endpoints of the edge.

### 2.3 Method used to identify new candidate chemicals

As mentioned in Section 2.2, interactive chemicals may share common functions. Specifically, interactive chemicals with high scores have a higher likelihood of sharing common functions than those with low scores. In view of this, in the constructed network in Section 2.2, chemicals occurring on the shortest path connecting two known lung cancer-related chemicals may have some functions shared by the known chemicals. Thus, we used Dijkstra’s algorithm [45], implemented in a graph theory software package of Maple 14 (http://www.maplesoft.com/), to search all the shortest paths connecting any pair of known chemicals related to lung cancer and collected all chemicals occurring in at least one path as inner nodes. These newly discovered chemicals were termed candidate chemicals. Additionally, we counted the number of paths containing each candidate chemical as an inner node and defined this value as betweenness. In fact, betweenness indicates the direct and indirect relationship of the candidate chemicals and known chemicals [46].

Furthermore, some chemicals may have a special position in the constructed network (*i*.*e*., these chemicals may always occur and receive high betweenness), even if we randomly selected some chemicals to search shortest paths connecting any pair of them. However, these chemicals have weak associations with lung cancer. To exclude this class of chemicals, a randomization test was executed as follows. We randomly constructed 500 chemical sets that had sizes equal to that of the set consisting of known chemicals. Then, for each set, all the shortest paths connecting any pair of chemicals in the set were found, and the betweenness of each candidate chemical was determined. Finally, we calculated the permutation FDR of each candidate chemical, which was defined as “the number of chemical sets in which the betweenness was higher than that for the known chemical set”/500. In fact, the permutation FDR can further measure the associations between candidate chemicals and lung cancer. Specifically, low permutation FDR of a candidate chemical indicates that its betweenness for the known chemical set is higher than or equal to those for the most randomly constructed chemical sets and implies that this candidate chemical is specific to lung cancer. High permutation FDR of a candidate chemical indicates that its betweenness for the known chemical set is smaller than those of the most randomly constructed chemical sets, suggesting that this candidate chemical is the general hub of the constructed network and not specific to lung cancer. Therefore, we selected candidate chemicals with permutation FDRs less than 0.05, which is often used as the cutoff of traditional significance level of the test.

### 2.4 Further selection by linking the candidate and lung cancer related chemicals

After executing the method mentioned in Section 2.3, some candidate chemicals for NSCLC and SCLC were extracted from the network constructed in Section 2.2. In this section, a further method was given to measure the relationship between each candidate chemical and lung cancer, thereby selecting candidate chemicals that have core associations with lung cancer. As mentioned above, interactive chemicals may share common functions [29,31,43]. However, chemicals with similar structures always have similar functions [47]. Therefore, we measured the associations between candidate chemicals and lung cancer based on the following two points: (1) chemical-chemical interactions between candidate chemicals and lung cancer-related chemicals; (2) chemical structure similarities between candidate chemicals and lung cancer-related chemicals.

For a candidate chemical *c* of NSCLC or SCLC, its maximum interaction score can be computed by:

$$Q_{NSCLC}^{i}(c)=\max\{S_{i}(c,c')|c'\in {\text{S}}_{\text{NSCLC}}\}$$ (1)

$$Q_{SCLC}^{i}(c)=\max\{S_{i}(c,c')|c'\in {\text{S}}_{\text{SCLC}}\}$$ (2)

It can be observed that high $Q_{NSCLC}^{i}(c)$ or high $Q_{SCLC}^{i}(c)$ indicates that the candidate chemical *c* is an interactive chemical of a NSCLC-related chemical or SCLC-related chemical with a high score, implying the candidate chemical *c* is closely related to NSCLC or SCLC. Here, we selected 900 as a threshold (*i*.*e*., candidate chemicals with maximum interaction score higher than or equal to 900 were selected) because 900 is set to be the threshold of the highest confidence level in STITCH.

Moreover, we also measured the relationships between candidate chemicals and lung cancer according to their structures. SMILES (Simplified Molecular Input Line Entry System) [48] is one of the most well-known chemical representation systems. Based on this type of representation and a particular fingerprint, a similarity score can be calculated to measure the structure similarity of two chemicals, which is given by Tanimoto coefficient (Tc) [49], in which chemicals that are identical have a Tc of 1.0, and compounds that are dissimilar have a Tc of 0. Here, FP2 fingerprint and Open Babel 2.3.2 [50] was used for pairwise Tc calculation. For formulation, let *S*_*s*_(*c*_1_, *c*_2_) be the similarity score of chemicals *c*_1_ and *c*_2_. Then, similar to **Eqs 1** and **2**, the maximum similarity score of a candidate chemical *c* of NSCLC or SCLC was calculated by

$$Q_{NSCLC}^{s}(c)=\max\{S_{s}(c,c')|c'\in {\text{S}}_{\text{NSCLC}}\}$$ (3)

$$Q_{SCLC}^{s}(c)=\max\{S_{s}(c,c')|c'\in {\text{S}}_{\text{SCLC}}\}$$ (4)

Similarly, high $Q_{NSCLC}^{s}(c)$ or high $Q_{SCLC}^{s}(c)$ indicates a close relationship between *c* and NSCLC or SCLC. Here, we selected 0.4 as a threshold (*i*.*e*., candidate chemicals with maximum similarity score higher than or equal to 0.4 were selected) because this value typically indicates that two chemical compounds share similar core substructures. Additionally, a Tc cutoff of 0.35–0.45 has also been frequently used for scaffold hopping and hit identification in computational drug design studies [51].

In summary, the candidate chemicals obtained by the method mentioned in Section 2.3 were further filtered by selecting chemicals with maximum interaction scores greater than or equal to 900 or maximum similarity scores greater than or equal to 0.4. The remaining candidate chemicals are deemed to have strong associations with lung cancer and termed significant candidate chemicals.

## Results and Discussion

### 3.1 Candidate chemicals for NSCLC and SCLC

**Fig 1:**
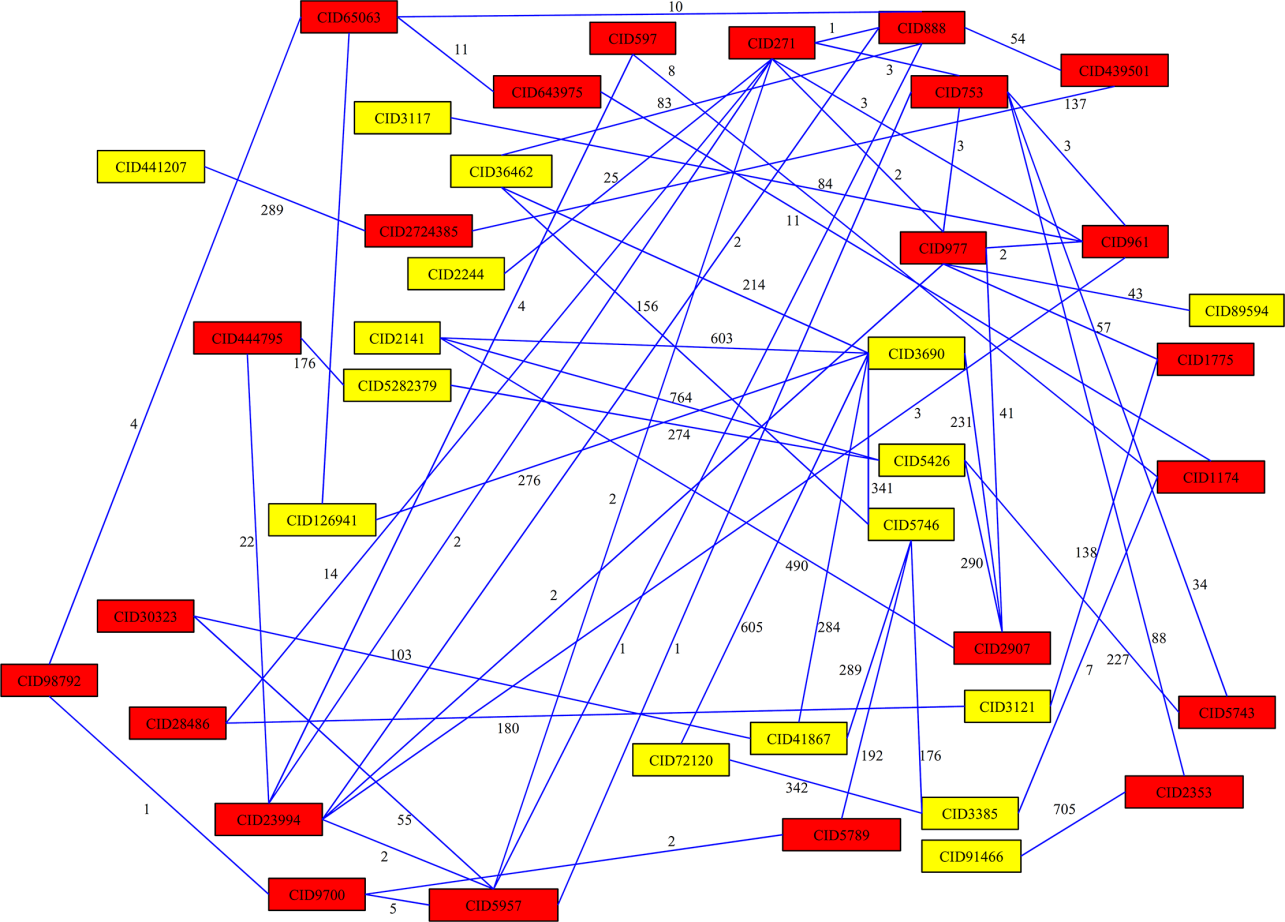
120 shortest paths connecting 16 NSCLC-related chemicals, which were obtained by applying Dijkstra’s algorithm in the constructed network. Yellow rectangles represent 16 NSCLC-related chemicals, and red rectangles represent 23 other chemicals involved in these 120 shortest paths. Numbers on edges represent edge weights in the network.

For NSCLC, we examined the shortest paths connecting any pair of the 16 known NSCLC-related chemicals. We obtained 120 shortest paths (see S1 Table for details), which are illustrated in **Fig 1**. It can be seen from **Fig 1** that 23 other chemicals were involved in these paths beyond the 16 NSCLC-related chemicals. These 23 chemicals were selected as candidate chemicals for NSCLC, which are listed **Table 2**. To exclude false discoveries, a randomization test was executed by calculating the permutation FDR for each candidate chemical, which is listed in column 5 of **Table 2**. We selected 0.05 as the threshold (*i*.*e*., only chemicals with permutation FDRs smaller than 0.05 were considered), thereby excluding three chemicals (see chemicals labeled with ‘c’ in **Table 2**): oxygen, adenosine triphosphate, hydroxyl radicals, and obtaining 20 candidate chemicals for NSCLC (see the first 20 chemicals in **Table 2**).

**Table 2:** Detailed information of 23 candidate chemicals for NSCLC.

Row number	PubChem ID	Name	Betweenness	Permutation FDR	Maximum interaction score	Maximum similarity score	Supporting reference
1	CID1174 ^a^	Uracil	34	<0.002	993	0.333	[52]
2	CID888 ^a^	Magnesium Ion	32	0.004	917	0	[53]
3	CID271 ^a^	Calcium Ion	36	0.008	975	0	[54,55]
4	CID444795	Tretinoin	14	<0.002	824	1	---
5	CID23994	Zinc	44	<0.002	940	0	---
6	CID643975	Flavin-Adenine Dinucleotide	2	0.032	900	0.102	---
7	CID439501	Ouabain	15	<0.002	549	0.449	---
8	CID2724385	Digoxin	15	<0.002	711	0.808	---
9	CID65063	2'-Deoxyuridylic Acid	14	0.006	959	0.138	---
10	CID753	Glycerol	28	0.036	925	0.167	---
11	CID597 ^b^	Cytosine	32	<0.002	762	0.182	---
12	CID2353 ^b^	Berberine	15	<0.002	295	0.179	---
13	CID2907 ^b^	Cyclophosphamide	22	<0.002	866	0.283	---
14	CID28486 ^b^	Lithium Ion	12	0.002	820	0	---
15	CID30323 ^b^	Daunorubicin	13	0.002	897	0.855	---
16	CID5743 ^b^	Dexamethasone	12	0.004	773	0.196	---
17	CID5789 ^b^	Thymidine	1	0.004	808	0.15	---
18	CID98792 ^b^	Dihydrofolate	5	0.01	844	0.352	---
19	CID9700 ^b^	Thymidine Monophosphate	5	0.036	724	0.141	---
20	CID1775 ^b^	Phenytoin	3	0.044	862	0.205	---
21	CID977 ^c^	Oxygen	34	0.142	---	---	---
22	CID5957 ^c^	Adenosine Triphosphate	27	0.17	---	---	---
23	CID961 ^c^	Hydroxyl Radicals	15	0.198	---	---	---

a: These chemicals were reported to be related to NSCLC in previous studies.

b: These chemicals were excluded by further selection because their maximum interaction scores were smaller than 900 and their maximum similarity scores were smaller than 0.4.

c: These chemicals were excluded by a randomization test because their permutation FDRs were equal to or larger than 0.05.

**Fig 2:**
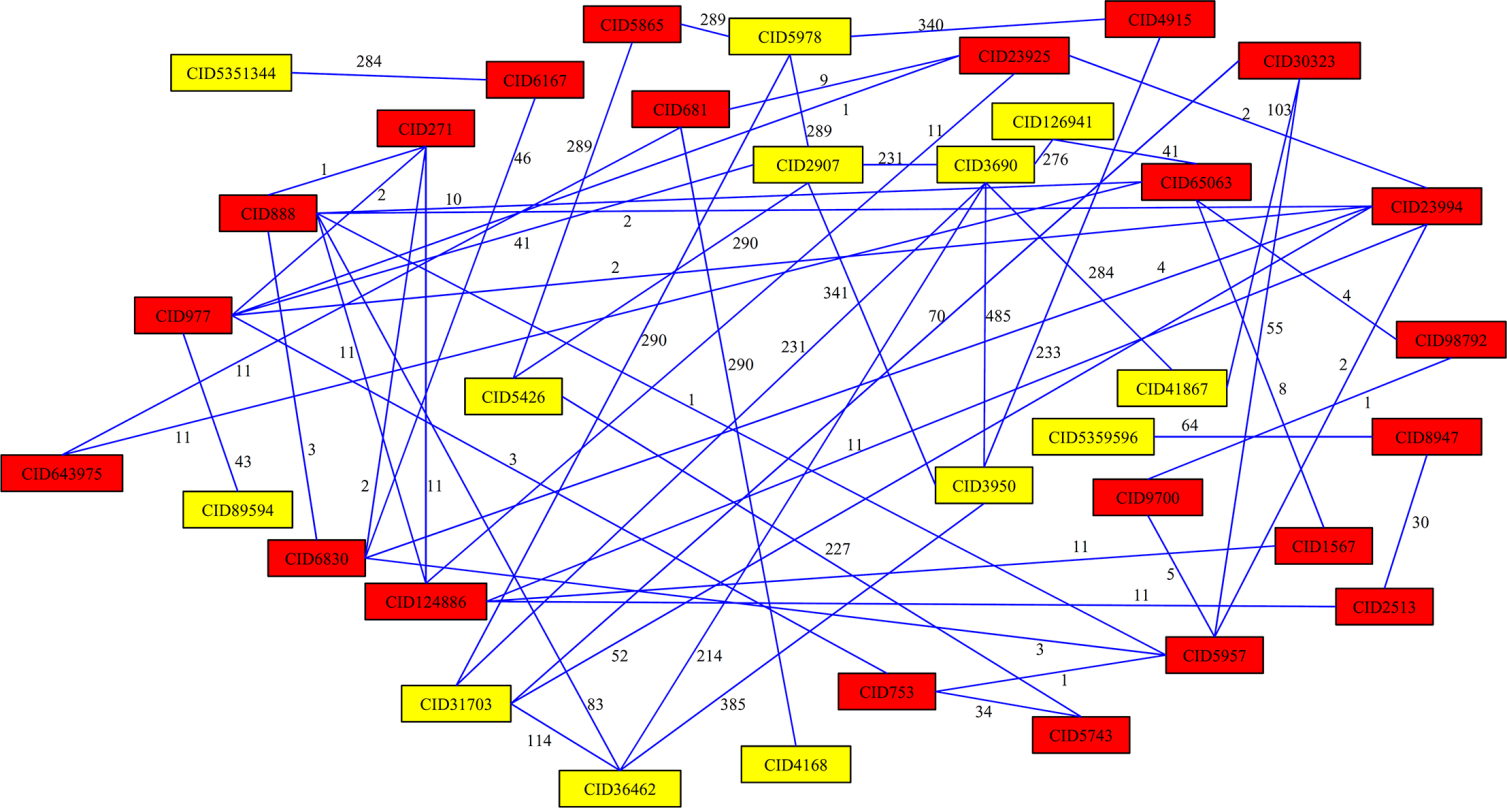
78 shortest paths connecting 13 SCLC-related chemicals, which were obtained by applying Dijkstra’s algorithm in the constructed network. Yellow rectangles represent 13 NSCLC-related chemicals, and red rectangles represent 22 other chemicals involved in these 78 shortest paths. Numbers on edges represent edge weights in the network.

Following the same procedures, 78 shortest paths (see S2 Table for details) connecting any pair of 13 known SCLC-related chemicals were obtained in the weighted network, which are illustrated in **Fig 2**. A total of 22 other chemicals were also involved in one of these paths beyond the 13 SCLC-related chemicals; these 22 chemicals were selected as candidate chemicals for SCLC. These candidate chemicals are listed in **Table 3**. Similarly, these candidate chemicals were filtered by a randomization test, thereby calculating the permutation FDR for each candidate chemical, which is listed in in column 5 of **Table 3**. Similar to NSCLC, we also selected 0.05 as the threshold. Thus, five chemicals (see chemicals labeled with ‘c’ in **Table 3**): magnesium, zinc, calcium, glycerol, adenosine triphosphate were excluded, and 17 candidate chemicals remained (see the first 17 chemicals in **Table 3**).

**Table 3:** Detailed information of 22 candidate chemicals for SCLC.

Row number	PubChem ID	Name	Betweenness	Permutation FDR	Maximum interaction score	Maximum similarity score	Supporting reference
1	CID977 ^a^	Oxygen	34	0.024	959	0.034	[85,86]
2	CID30323	Daunorubicin	11	0.004	930	0.855	---
3	CID5865	Prednisone	1	<0.002	903	0.170	---
4	CID8947	Monomethylarsonic Acid	12	<0.002	936	0.042	---
5	CID1567	Mercaptoethanol	1	0.004	921	0.039	---
6	CID65063	2'-Deoxyuridylic acid	11	0.004	959	0.138	---
7	CID2513 ^b^	Cacodylic Acid	12	<0.002	659	0.057	---
8	CID4915 ^b^	Procarbazine	1	<0.002	864	0.219	---
9	CID6167 ^b^	Colchicine	12	<0.002	716	0.176	---
10	CID124886 ^b^	Glutathione	12	<0.002	0	0.188	---
11	CID6830 ^b^	Guanosine Triphosphate	12	0.002	327	0.112	---
12	CID681 ^b^	Dopamine	12	0.01	802	0.163	---
13	CID5743 ^b^	Dexamethasone	8	0.01	773	0.196	---
14	CID98792 ^b^	Dihydrofolate	3	0.02	844	0.352	---
15	CID23925 ^b^	Iron	16	0.024	542	0	---
16	CID9700 ^b^	Thymidine Monophosphate	3	0.032	724	0.135	---
17	CID643975 ^b^	Flavin-Adenine Dinucleotide	1	0.034	0	0.096	---
18	CID888 ^c^	Magnesium	15	0.058	---	---	---
19	CID23994 ^c^	Zinc	12	0.122	---	---	---
20	CID271 ^c^	Calcium	13	0.158	---	---	---
21	CID753 ^c^	Glycerol	9	0.228	---	---	---
22	CID5957 ^c^	Adenosine Triphosphate	15	0.28	---	---	---

a: These chemicals were reported to be related to SCLC in previous studies.

b: These chemicals were excluded by further selection because their maximum interaction scores were smaller than 900 and their maximum similarity scores were smaller than 0.4.

c: These chemicals were excluded by a randomization test because their permutation FDRs were equal to or larger than 0.05.

### 3.2 Significant candidate chemicals for NSCLC and SCLC

According to the procedures described in Section 2.4, for each of 20 candidate chemicals of NSCLC, we calculated the maximum interaction score (cf. **Eq 1**) and maximum similarity score (cf. **Eq 3**); these values are listed in column 6 and 7 of **Table 2,** respectively. After checking these scores, ten candidate chemicals (see chemicals labeled with ‘b’ in **Table 2**) were excluded because their maximum interaction scores were smaller than 900 and maximum similarity scores were smaller than 0.4. Ten candidate chemicals remained (see the first ten chemicals in **Table 2**), which were deemed to be highly related to NSCLC, and these compounds were termed significant candidate chemicals for NSCLC.

For SCLC, the maximum interaction score and maximum similarity score of each candidate chemical were calculated by **Eq 2** and **Eq 4**, respectively. These scores are listed in column 6 and 7 of **Table 3**, respectively. Six candidate chemicals received maximum interaction scores greater than or equal to 900 or maximum similarity scores greater than or equal to 0.4, and eleven chemicals (see chemicals labeled with ‘b’ in **Table 3**) were excluded. The remaining six candidate chemicals were deemed to have strong associations with SCLC and termed significant candidate chemicals for SCLC.

### 3.3 Analysis of significant candidate chemicals for NSCLC

In this study, we identified ten new candidate chemicals related to NSCLC (see the first ten chemicals in **Table 2**). Of these ten candidate chemicals, three chemicals: uracil, magnesium ion, calcium ion (see the first three chemicals in **Table 2**) have been reported to be related to NSCLC in some previous studies [52,53,54,55]. For the remaining seven candidate chemicals, five were found to have associations with NSCLC according to their currently known functions (listed in rows 4–8 of **Table 2**). The following paragraphs provide a detailed discussion of the associations between these chemicals and NSCLC.

#### Tretinoin

This chemical was identified as a significant candidate chemical for NSCLC (see row 4 of **Table 2**). Tretinoin, or all-trans-retinoic acid (ATRA), is derived from vitamin A and plays an important role in the regulation of gene expression. It has been widely used in the treatment of acute promyelocytic leukemia (APL) because ATRA inhibits the growth of myeloma cells by restraining both interleukin 6 (IL-6) and its receptor (IL-6R) [56,57]. Moreover, it was recently reported that the proliferation of lung fibroblasts induced by irradiation is inhibited by ATRA, also through the suppression of the cytokines IL-6 and IL-6R [58]. TGF-β and PDGF are also potential targets of ATRA [59]. There have been attempts to use ATRA as a chemotherapeutic for the treatment of lung cancer [60,61]. However, the effects of ATRA on tumorigenesis are complex. In A549 cells, a human lung adenocarcinoma cell line, ATRA upregulates the expression of VEGF, which gives rise to angiogenesis and cancer growth [62,63]. If the induced VEGF can be countered, ATRA is a promising drug for lung cancer therapy.

#### Zinc

This chemical was identified as a significant candidate chemical for NSCLC (see row 5 of **Table 2**). Zinc (molecular formula: Zn) is a metallic element, which is required for over 300 enzymes and 2,000 transcription factors involved in many enzymatic and metabolic functions [64]. In our study, zinc had a betweenness score of 44 and a maximum link to known compounds score of 940, indicating a significant relationship with NSCLC. It has been observed that a zinc deficiency may be related to the increased risk of cancer in epidemiologic studies [65]. Immune function such as the activity of natural killer and cytolytic T cells is decreased in zinc deficiency [65]. The downregulation of IL-2 and IL-2 receptors may be due to the suppression of NF-kB caused by zinc deficiency [66]. Additionally, zinc deficiency gives rise to the excess production of ROS, which is an essential factor in tumorigenesis [65]. In head and neck cancer patients, the tumor size and stage were closely associated with zinc deficiency [67]. These adverse effects are reversible with zinc supplementation, suggesting that zinc supplementation may be an agent for lung cancer chemoprevention.

#### FAD

This chemical was identified as a significant candidate chemical for NSCLC (see row 6 of **Table 2**). Involved in many essential reactions, Flavin adenine dinucleotide (FAD) is a redox cofactor with two redox states: FAD and FADH_2_. Our data reveals that the FAD has a betweenness score of 2 and a maximum link to known compounds score of 900. In PCa (prostate cancer) cells, the acetyl derivatives of spermidine and spermine are oxidized by acetyl polyamine oxidase (APAO), excess ROS are produced, and FAD is released [68,69]. The concentration of FAD was increased by APAO enhancive activity within cells due to the FADH_2_ to FAD conversion [70,71,72]. The function of p53, a key tumor suppressor, is to affect MDM2-independent, NADH quinone oxidoreductase 1-mediated protein degradation, which is likely due to the imbalance of FAD/NAD in vitro [73]. The role of FAD in cancer is unclear and requires further research.

#### Ouabain

This chemical was identified as a significant candidate chemical for NSCLC (see row 7 of **Table 2**). Ouabain is a cardiac glycoside, which has been identified as a human hormone. Many studies show that ouabain plays an important role in cancer and possesses anti-tumor activity [74,75]. Ouabain has been found to mediate cell apoptosis through TRAIL (necrosis factor-related apoptosis-inducing legend) [76] and enhance lung cancer cell detachment [77]. In lung cancer cell lines, ouabain suppressed metastasis by regulating integrin, which caused resistance to chemotherapeutic agents [78,79]. Ouabain is also a Na+, K+-ATPase inhibitor that may mediate its anti-tumor function [80]. In our study, a close relationship was observed between ouabain and NSCLC.

#### Digoxin

This chemical was identified as a significant candidate chemical for NSCLC (see row 8 of **Table 2**). Digoxin, also known as 12-beta-hydroxydigitoxin, is a cardiac glycoside and has been used to treat heart-related diseases, but it may be toxic to heath. Digoxin is a known inhibitor of Na+/K+ ATPase and disrupts the balance in intracellular Ca^2+^ and Na^+^ concentrations [81], which may be the mechanism of digoxin-induced apoptosis. In the 549 cell line (the NSCLC cell line), the hypoxic conditions induced VEGF (Vascular endothelial growth factor) and NDRG1 (N-Myc downregulated gene 1) overexpression, and tumor cell proliferation was suppressed by digoxin, likely through the inhibition of HIF1-α (hypoxia-inducible factor-1α) [82]. In a model of neuroblastoma mice, tumor growth was inhibited by digoxin [83]. In our study, digoxin has a betweenness score of 15 and is significantly associated with NSCLC. The above evidence indicates that digoxin is a potential chemotherapy drug for NSCLC patients. However, the dosage window between toxicity and therapy is small, and humans are more sensitive to the drug’s toxicity than mice [84], indicating that it must be carefully tested clinically.

For the remaining two significant candidate chemicals (2'-deoxyuridylic acid, Glycerol), we could not find any literature reporting associations between them and NSCLC. However, their possibility cannot be excluded. We list them in rows 9–10 of **Table 2** and hope that they may be further studied in the context of NSCLC.

### 3.4 Analysis of significant candidate chemicals for SCLC

Similar to NSCLC, we identified six new candidate chemicals related to SCLC. Of these six significant candidate chemicals, one chemical, oxygen (see row 1 of **Table 3**), has been reported to be related to SCLC in some previous studies [85,86]. Among the remaining five significant candidate chemicals, three were found to have associations with SCLC (listed in rows 2–4 of **Table 3**). The following paragraphs provide a detailed discussion of the associations between these chemicals and SCLC.

#### Daunorubicin

This chemical was identified as a significant candidate chemical for SCLC (see row 2 of **Table 3**). Daunorubicin, or Daunomycin (DAUD), is an aminoglycoside antineoplastic, isolated from *Streptomyces peucetius* and other bacteria. DAUD is used to treat various types of cancer because of its antineoplastic effects [87,88]. However, due to side effects, its clinical application is limited. The mechanism of antineoplastic and cytotoxic effects is not clear. It has been speculated that it may be involved in DNA and RNA synthesis (DNA damage through interference with topoisomerase II, cell apoptosis and iron channel balance) [89,90,91]. The aldo-keto reductases (AKRs) and carbonyl reductases (CBRs), which have different enzymatic activity in DAUD-stimulated cell lines, have been implicated in the metabolism of DAUD [92]. AKRs and CBRs play essential roles in various biological functions in lung cancer. Our study revealed that DAUD is closely associated with both NSCLC and SCLC. As a widely used antitumor drug, DAUD is a potential drug to treat lung cancer. Considering the side effects of DAUD, more studies are needed on the appropriate dosage and the mechanism underlying the antineoplastic and cytotoxicity effects.

#### Prednisone

This chemical was identified as a significant candidate chemical for SCLC (see row 3 of **Table 3**). Prednisone, also known as meticorten and short for CPR, is a synthetic glucocorticoid obtained from cortisone. CPR is utilized as an agent of multi-drug therapy for the treatment of some tumors [93]. The combination drug therapy of mitoxantrone and low-dose prednisone had fewer side effects and an improved quality of life compared with patients taking CPR alone [94,95]. In metastatic castration-resistant prostate cancer (mCRPC) patients, the combination therapy of prednisone, azacitidine and docetaxel with growth factor (GF) support is effective [96], although the mechanism responsible for its anti-tumor and cytotoxicity activity is unclear. In our study, CPR was closely associated with SCLC and may be an effective chemotherapy drug for lung cancer.

#### Monomethylarsonic Acid

This chemical was identified as a significant candidate chemical for SCLC (see row 4 of **Table 3**). Monomethylarsonic acid (MMA V) is synonymous with Methylarsonous acid (MMA III) in Medical Subject Heading (MeSH). MMA V is the methylated metabolite of inorganic arsenic (iAs) and is reduced to MMA III [97]. MMA III is the methylated metabolite of inorganic arsenic (iAs), both of which are potential carcinogenic materials in rodents [98,99,100]. In our study, MMA III showed a betweenness score of 12 and a maximum link to known compound score of 936, which indicated a close relationship with NSCLC. In various cell lines including skin, lung, liver, prostate, and kidney, malignant transformation was induced by iAs [101,102,103,104,105,106], and in urinary bladder cell lines, the malignant transformation of cells can be caused by MMA III [107,108]. It has been shown that iAs and MMA III can induce the generation of ROS and ODD (oxidative DNA damage), both of which are involved in carcinogenesis [109,110,111]. Oxidative damage is not the only effect of arsenicals; arsenic can also deplete the expression of PTEN, a tumor suppressor gene [99, 112,113], leading to further genomic instability [114]. Some studies suggest that MMA III may be even more cytotoxic than iAs [115]. As discussed above, MMA III and iAs are important carcinogens requiring further research.

For the remaining two significant candidate chemicals (mercaptoethanol and 2'-deoxyuridylic acid), no literature reported that they were associated with SCLC. However, we cannot confirm that they have no associations with SCLC (*i*.*e*., they may still be related to SCLC). We list them in rows 5–6 of **Table 3** and hope that they may further studied in the context of SCLC.

### 3.5 Analysis of other candidate chemicals

Some chemicals with weak associations with NSCLC-related or SCLC-related chemicals are possible putative anti-carcinogenesis drugs. There are few studies regarding their roles in lung cancers, but there is evidence indicating that they have antitumor effects in other cancers. This finding suggests that they may be putative attractive antineoplastic drugs for NSCLC/SCLC. Two of them are discussed below.

#### Berberine

This chemical is related to NSCLC (see row 12 of **Table 2**). Berberine (BBR), or Umbellatine, is a member of the isoquinoline alkaloids, which are found in some medicinal plants such as Rhizoma Coptidis and *Coptis chinensis* [116]. Initially, due to its antibacterial properties, BBR was widely used to treat bacterial and fungal infections. It also has an antineoplastic effect in various cancers including leukemia and large intestine carcinoma [117,118]. In breast cancer, apoptosis of tumor cells is induced by TRAIL (tumor necrosis factor related apoptosis-inducing ligand), which is enhanced by BBR [119]. The AP-1 signaling pathway and the transcription factors binding to the CCND1 (cyclin D1) AP-1 motif were suppressed by BBR in PG cells (human lung carcinoma cell line), which may be an important anti-cancer mechanism [120]. In A549 lung cancer cells, TGF-β induced EMT is inhibited by BBR, revealing a potential mechanism for the anti-invasion and anti-metastasis effects [121]. Additionally, BBR has low toxicity in normal cells, which indicates that BBR is a putative attractive antineoplastic drug [122,123,124].

#### Colchicine

This chemical is related to SCLC (see row 9 of **Table 3**). Colchicine, also known as Colcin, is isolated from *Colchicum autumnae*, which is used for the treatment of gout and Mediterranean fever [125,126]. Colchicine has strong tubulin binding capacity, which perturbs microtubule assembly, therefore limiting its clinical application. One marked characteristic of cancer cells is their high-rate of mitosis rendering them more sensitive to colchicine. In fact, the growth of tumor cells in hepatocellular carcinoma (HCC) is inhibited by colchicine with few side effects [127]. The expression of MX dynamin-like GTPase 1 (MX1) and TGFB2 are upregulated by colchicine in these HCC cells, which may be one of the mechanisms of its antineoplastic function [128]. Although colchicine shows great promise as a chemotherapeutic for lung cancer, the curative effect and clinical dose are not yet clear. Furthermore, more research is needed to develop better drug delivery strategies, which directly target the cancer cells and reduce chemotherapeutic toxicity.

## Conclusions

In this study, we proposed a variation on an existing computational method to identify new candidate chemicals related to non-small lung cancer and small-cell lung cancer. According to the literature, some newly discovered chemicals have strong associations with the biological process of lung cancer. Future research is required to replicate and validate the new findings in this study and to shed new light on the study of lung cancer and other diseases.

## Supporting Information

S1 Table The information of 120 shortest paths connecting 16 NSCLC-related chemicals. (DOCX)

S2 Table The information of 78 shortest paths connecting 13 SCLC-related chemicals. (DOCX)
