# Supplementary material for: The Use of Chemical-Chemical Interaction and Chemical Structure to Identify New Candidate Chemicals Related to Lung Cancer
Source: PLoS One. 2015 Jun 5;10(6):e0128696. doi: 10.1371/journal.pone.0128696 (PMC4457841; doi:10.1371/journal.pone.0128696)
Supplement: S1 Table — (DOCX) [file pone.0128696.s001.docx]

**S1 Table.** The information of 120 shortest paths connecting 16 NSCLC-related chemicals

| **Weight of shortest path** | **Path** |
| --- | --- |
| 558 | CID2141 CID2907 CID977 CID271 CID2244 |
| 617 | CID2141 CID2907 CID977 CID961 CID3117 |
| 726 | CID2141 CID2907 CID977 CID1775 CID3121 |
| 552 | CID2141 CID2907 CID977 CID23994 CID597 CID1174 CID3385 |
| 603 | CID2141 CID3690 |
| 764 | CID2141 CID5426 |
| 728 | CID2141 CID2907 CID977 CID23994 CID597 CID1174 CID3385 CID5746 |
| 617 | CID2141 CID2907 CID977 CID271 CID888 CID36462 |
| 693 | CID2141 CID2907 CID977 CID23994 CID5957 CID30323 CID41867 |
| 894 | CID2141 CID2907 CID977 CID23994 CID597 CID1174 CID3385 CID72120 |
| 574 | CID2141 CID2907 CID977 CID89594 |
| 1327 | CID2141 CID2907 CID977 CID753 CID2353 CID91466 |
| 585 | CID2141 CID2907 CID977 CID271 CID888 CID65063 CID126941 |
| 1014 | CID2141 CID2907 CID977 CID271 CID888 CID439501 CID2724385 CID441207 |
| 731 | CID2141 CID2907 CID977 CID23994 CID444795 CID5282379 |
| 112 | CID2244 CID271 CID961 CID3117 |
| 219 | CID2244 CID271 CID28486 CID3121 |
| 46 | CID2244 CID271 CID23994 CID597 CID1174 CID3385 |
| 299 | CID2244 CID271 CID977 CID2907 CID3690 |
| 289 | CID2244 CID271 CID753 CID5743 CID5426 |
| 222 | CID2244 CID271 CID23994 CID597 CID1174 CID3385 CID5746 |
| 109 | CID2244 CID271 CID888 CID36462 |
| 185 | CID2244 CID271 CID5957 CID30323 CID41867 |
| 388 | CID2244 CID271 CID23994 CID597 CID1174 CID3385 CID72120 |
| 70 | CID2244 CID271 CID977 CID89594 |
| 821 | CID2244 CID271 CID753 CID2353 CID91466 |
| 77 | CID2244 CID271 CID888 CID65063 CID126941 |
| 506 | CID2244 CID271 CID888 CID439501 CID2724385 CID441207 |
| 225 | CID2244 CID271 CID23994 CID444795 CID5282379 |
| 281 | CID3117 CID961 CID271 CID28486 CID3121 |
| 106 | CID3117 CID961 CID23994 CID597 CID1174 CID3385 |
| 358 | CID3117 CID961 CID977 CID2907 CID3690 |
| 348 | CID3117 CID961 CID753 CID5743 CID5426 |
| 282 | CID3117 CID961 CID23994 CID597 CID1174 CID3385 CID5746 |
| 171 | CID3117 CID961 CID271 CID888 CID36462 |
| 246 | CID3117 CID961 CID753 CID5957 CID30323 CID41867 |
| 448 | CID3117 CID961 CID23994 CID597 CID1174 CID3385 CID72120 |
| 129 | CID3117 CID961 CID977 CID89594 |
| 880 | CID3117 CID961 CID753 CID2353 CID91466 |
| 139 | CID3117 CID961 CID271 CID888 CID65063 CID126941 |
| 568 | CID3117 CID961 CID271 CID888 CID439501 CID2724385 CID441207 |
| 285 | CID3117 CID961 CID23994 CID444795 CID5282379 |
| 215 | CID3121 CID28486 CID271 CID23994 CID597 CID1174 CID3385 |
| 467 | CID3121 CID1775 CID977 CID2907 CID3690 |
| 458 | CID3121 CID28486 CID271 CID753 CID5743 CID5426 |
| 391 | CID3121 CID28486 CID271 CID23994 CID597 CID1174 CID3385 CID5746 |
| 278 | CID3121 CID28486 CID271 CID888 CID36462 |
| 354 | CID3121 CID28486 CID271 CID5957 CID30323 CID41867 |
| 557 | CID3121 CID28486 CID271 CID23994 CID597 CID1174 CID3385 CID72120 |
| 238 | CID3121 CID1775 CID977 CID89594 |
| 990 | CID3121 CID28486 CID271 CID753 CID2353 CID91466 |
| 246 | CID3121 CID28486 CID271 CID888 CID65063 CID126941 |
| 675 | CID3121 CID28486 CID271 CID888 CID439501 CID2724385 CID441207 |
| 394 | CID3121 CID28486 CID271 CID23994 CID444795 CID5282379 |
| 293 | CID3385 CID1174 CID597 CID23994 CID977 CID2907 CID3690 |
| 283 | CID3385 CID1174 CID597 CID23994 CID5957 CID753 CID5743 CID5426 |
| 176 | CID3385 CID5746 |
| 104 | CID3385 CID1174 CID597 CID23994 CID888 CID36462 |
| 179 | CID3385 CID1174 CID597 CID23994 CID5957 CID30323 CID41867 |
| 342 | CID3385 CID72120 |
| 64 | CID3385 CID1174 CID597 CID23994 CID977 CID89594 |
| 815 | CID3385 CID1174 CID597 CID23994 CID5957 CID753 CID2353 CID91466 |
| 70 | CID3385 CID1174 CID643975 CID65063 CID126941 |
| 501 | CID3385 CID1174 CID597 CID23994 CID888 CID439501 CID2724385 CID441207 |
| 217 | CID3385 CID1174 CID597 CID23994 CID444795 CID5282379 |
| 521 | CID3690 CID2907 CID5426 |
| 341 | CID3690 CID5746 |
| 214 | CID3690 CID36462 |
| 284 | CID3690 CID41867 |
| 605 | CID3690 CID72120 |
| 315 | CID3690 CID2907 CID977 CID89594 |
| 1068 | CID3690 CID2907 CID977 CID753 CID2353 CID91466 |
| 276 | CID3690 CID126941 |
| 755 | CID3690 CID2907 CID977 CID271 CID888 CID439501 CID2724385 CID441207 |
| 472 | CID3690 CID2907 CID977 CID23994 CID444795 CID5282379 |
| 459 | CID5426 CID5743 CID753 CID5957 CID23994 CID597 CID1174 CID3385 CID5746 |
| 346 | CID5426 CID5743 CID753 CID5957 CID888 CID36462 |
| 420 | CID5426 CID5743 CID753 CID5957 CID30323 CID41867 |
| 625 | CID5426 CID5743 CID753 CID5957 CID23994 CID597 CID1174 CID3385 CID72120 |
| 307 | CID5426 CID5743 CID753 CID977 CID89594 |
| 1054 | CID5426 CID5743 CID753 CID2353 CID91466 |
| 313 | CID5426 CID5743 CID753 CID5957 CID9700 CID98792 CID65063 CID126941 |
| 743 | CID5426 CID5743 CID753 CID5957 CID888 CID439501 CID2724385 CID441207 |
| 274 | CID5426 CID5282379 |
| 156 | CID5746 CID36462 |
| 289 | CID5746 CID41867 |
| 518 | CID5746 CID3385 CID72120 |
| 240 | CID5746 CID3385 CID1174 CID597 CID23994 CID977 CID89594 |
| 991 | CID5746 CID3385 CID1174 CID597 CID23994 CID5957 CID753 CID2353 CID91466 |
| 240 | CID5746 CID5789 CID9700 CID98792 CID65063 CID126941 |
| 677 | CID5746 CID3385 CID1174 CID597 CID23994 CID888 CID439501 CID2724385 CID441207 |
| 393 | CID5746 CID3385 CID1174 CID597 CID23994 CID444795 CID5282379 |
| 242 | CID36462 CID888 CID5957 CID30323 CID41867 |
| 446 | CID36462 CID888 CID23994 CID597 CID1174 CID3385 CID72120 |
| 129 | CID36462 CID888 CID271 CID977 CID89594 |
| 878 | CID36462 CID888 CID5957 CID753 CID2353 CID91466 |
| 134 | CID36462 CID888 CID65063 CID126941 |
| 563 | CID36462 CID888 CID439501 CID2724385 CID441207 |
| 283 | CID36462 CID888 CID23994 CID444795 CID5282379 |
| 521 | CID41867 CID30323 CID5957 CID23994 CID597 CID1174 CID3385 CID72120 |
| 205 | CID41867 CID30323 CID5957 CID753 CID977 CID89594 |
| 952 | CID41867 CID30323 CID5957 CID753 CID2353 CID91466 |
| 209 | CID41867 CID30323 CID5957 CID9700 CID98792 CID65063 CID126941 |
| 639 | CID41867 CID30323 CID5957 CID888 CID439501 CID2724385 CID441207 |
| 358 | CID41867 CID30323 CID5957 CID23994 CID444795 CID5282379 |
| 406 | CID72120 CID3385 CID1174 CID597 CID23994 CID977 CID89594 |
| 1157 | CID72120 CID3385 CID1174 CID597 CID23994 CID5957 CID753 CID2353 CID91466 |
| 412 | CID72120 CID3385 CID1174 CID643975 CID65063 CID126941 |
| 843 | CID72120 CID3385 CID1174 CID597 CID23994 CID888 CID439501 CID2724385 CID441207 |
| 559 | CID72120 CID3385 CID1174 CID597 CID23994 CID444795 CID5282379 |
| 839 | CID89594 CID977 CID753 CID2353 CID91466 |
| 97 | CID89594 CID977 CID271 CID888 CID65063 CID126941 |
| 526 | CID89594 CID977 CID271 CID888 CID439501 CID2724385 CID441207 |
| 243 | CID89594 CID977 CID23994 CID444795 CID5282379 |
| 845 | CID91466 CID2353 CID753 CID5957 CID9700 CID98792 CID65063 CID126941 |
| 1275 | CID91466 CID2353 CID753 CID5957 CID888 CID439501 CID2724385 CID441207 |
| 994 | CID91466 CID2353 CID753 CID5957 CID23994 CID444795 CID5282379 |
| 531 | CID126941 CID65063 CID888 CID439501 CID2724385 CID441207 |
| 251 | CID126941 CID65063 CID98792 CID9700 CID5957 CID23994 CID444795 CID5282379 |
| 680 | CID441207 CID2724385 CID439501 CID888 CID23994 CID444795 CID5282379 |
