# Supplementary material for: The Use of Chemical-Chemical Interaction and Chemical Structure to Identify New Candidate Chemicals Related to Lung Cancer
Source: PLoS One. 2015 Jun 5;10(6):e0128696. doi: 10.1371/journal.pone.0128696 (PMC4457841; doi:10.1371/journal.pone.0128696)
Supplement: S2 Table — (DOCX) [file pone.0128696.s002.docx]

**S2 Table.** The information of 78 shortest paths connecting 13 SCLC-related chemicals

| **Weight of shortest path** | **Path** |
| --- | --- |
| 231 | CID2907 CID3690 |
| 341 | CID2907 CID3950 |
| 341 | CID2907 CID977 CID23925 CID681 CID4168 |
| 290 | CID2907 CID5426 |
| 289 | CID2907 CID5978 |
| 95 | CID2907 CID977 CID23994 CID31703 |
| 127 | CID2907 CID977 CID271 CID888 CID36462 |
| 203 | CID2907 CID977 CID23994 CID5957 CID30323 CID41867 |
| 84 | CID2907 CID977 CID89594 |
| 95 | CID2907 CID977 CID271 CID888 CID65063 CID126941 |
| 375 | CID2907 CID977 CID271 CID6830 CID6167 CID5351344 |
| 158 | CID2907 CID977 CID23925 CID124886 CID2513 CID8947 CID5359596 |
| 485 | CID3690 CID3950 |
| 572 | CID3690 CID2907 CID977 CID23925 CID681 CID4168 |
| 521 | CID3690 CID2907 CID5426 |
| 520 | CID3690 CID2907 CID5978 |
| 231 | CID3690 CID31703 |
| 214 | CID3690 CID36462 |
| 284 | CID3690 CID41867 |
| 315 | CID3690 CID2907 CID977 CID89594 |
| 276 | CID3690 CID126941 |
| 606 | CID3690 CID2907 CID977 CID271 CID6830 CID6167 CID5351344 |
| 389 | CID3690 CID2907 CID977 CID23925 CID124886 CID2513 CID8947 CID5359596 |
| 682 | CID3950 CID2907 CID977 CID23925 CID681 CID4168 |
| 631 | CID3950 CID2907 CID5426 |
| 573 | CID3950 CID4915 CID5978 |
| 436 | CID3950 CID2907 CID977 CID23994 CID31703 |
| 385 | CID3950 CID36462 |
| 544 | CID3950 CID2907 CID977 CID23994 CID5957 CID30323 CID41867 |
| 425 | CID3950 CID2907 CID977 CID89594 |
| 436 | CID3950 CID2907 CID977 CID271 CID888 CID65063 CID126941 |
| 716 | CID3950 CID2907 CID977 CID271 CID6830 CID6167 CID5351344 |
| 499 | CID3950 CID2907 CID977 CID23925 CID124886 CID2513 CID8947 CID5359596 |
| 564 | CID4168 CID681 CID23925 CID977 CID753 CID5743 CID5426 |
| 630 | CID4168 CID681 CID23925 CID977 CID2907 CID5978 |
| 353 | CID4168 CID681 CID23925 CID23994 CID31703 |
| 386 | CID4168 CID681 CID23925 CID23994 CID888 CID36462 |
| 461 | CID4168 CID681 CID23925 CID23994 CID5957 CID30323 CID41867 |
| 343 | CID4168 CID681 CID23925 CID977 CID89594 |
| 353 | CID4168 CID681 CID643975 CID65063 CID126941 |
| 634 | CID4168 CID681 CID23925 CID977 CID271 CID6830 CID6167 CID5351344 |
| 415 | CID4168 CID681 CID23925 CID124886 CID2513 CID8947 CID5359596 |
| 578 | CID5426 CID5865 CID5978 |
| 316 | CID5426 CID5743 CID753 CID5957 CID23994 CID31703 |
| 346 | CID5426 CID5743 CID753 CID5957 CID888 CID36462 |
| 420 | CID5426 CID5743 CID753 CID5957 CID30323 CID41867 |
| 307 | CID5426 CID5743 CID753 CID977 CID89594 |
| 313 | CID5426 CID5743 CID753 CID5957 CID9700 CID98792 CID65063 CID126941 |
| 595 | CID5426 CID5743 CID753 CID5957 CID6830 CID6167 CID5351344 |
| 379 | CID5426 CID5743 CID753 CID5957 CID888 CID124886 CID2513 CID8947 CID5359596 |
| 290 | CID5978 CID31703 |
| 404 | CID5978 CID31703 CID36462 |
| 463 | CID5978 CID31703 CID30323 CID41867 |
| 373 | CID5978 CID2907 CID977 CID89594 |
| 384 | CID5978 CID2907 CID977 CID271 CID888 CID65063 CID126941 |
| 664 | CID5978 CID2907 CID977 CID271 CID6830 CID6167 CID5351344 |
| 447 | CID5978 CID2907 CID977 CID23925 CID124886 CID2513 CID8947 CID5359596 |
| 114 | CID31703 CID36462 |
| 173 | CID31703 CID30323 CID41867 |
| 97 | CID31703 CID23994 CID977 CID89594 |
| 105 | CID31703 CID23994 CID888 CID65063 CID126941 |
| 386 | CID31703 CID23994 CID6830 CID6167 CID5351344 |
| 168 | CID31703 CID23994 CID124886 CID2513 CID8947 CID5359596 |
| 242 | CID36462 CID888 CID5957 CID30323 CID41867 |
| 129 | CID36462 CID888 CID271 CID977 CID89594 |
| 134 | CID36462 CID888 CID65063 CID126941 |
| 416 | CID36462 CID888 CID6830 CID6167 CID5351344 |
| 199 | CID36462 CID888 CID124886 CID2513 CID8947 CID5359596 |
| 205 | CID41867 CID30323 CID5957 CID753 CID977 CID89594 |
| 209 | CID41867 CID30323 CID5957 CID9700 CID98792 CID65063 CID126941 |
| 491 | CID41867 CID30323 CID5957 CID6830 CID6167 CID5351344 |
| 275 | CID41867 CID30323 CID5957 CID888 CID124886 CID2513 CID8947 CID5359596 |
| 97 | CID89594 CID977 CID271 CID888 CID65063 CID126941 |
| 377 | CID89594 CID977 CID271 CID6830 CID6167 CID5351344 |
| 160 | CID89594 CID977 CID23925 CID124886 CID2513 CID8947 CID5359596 |
| 384 | CID126941 CID65063 CID98792 CID9700 CID5957 CID6830 CID6167 CID5351344 |
| 165 | CID126941 CID65063 CID1567 CID124886 CID2513 CID8947 CID5359596 |
| 448 | CID5351344 CID6167 CID6830 CID271 CID124886 CID2513 CID8947 CID5359596 |
